# Supplementary material for: From sequence to enzyme mechanism using multi-label machine learning
Source: BMC Bioinformatics. 2014 May 19;15:150. doi: 10.1186/1471-2105-15-150 (PMC4229970; doi:10.1186/1471-2105-15-150)
Supplement: Additional file 2 — Java code of ml2db. Additional file ml2db_code.tar.gz contains the Java source code to run the multi-label machine learning experiments and save the results to database. The code’s Javadoc is included. [file 1471-2105-15-150-S2.zip › additional file 2/ml2db/ml2db/doc/mulan/classifier/meta/ConstrainedKMeans.bucketInstance.html]

ConstrainedKMeans.bucketInstance


JavaScript is disabled on your browser.


- Overview
- Package
- Class
- Use
- Tree
- Deprecated
- Index
- Help

- Prev Class
- Next Class

- Frames
- No Frames

- All Classes

- Summary:
- Nested |
- Field |
- Constr |
- Method

- Detail:
- Field |
- Constr |
- Method


mulan.classifier.meta

## Class ConstrainedKMeans.bucketInstance

- java.lang.Object
- - mulan.classifier.meta.ConstrainedKMeans.bucketInstance

- All Implemented Interfaces:
  :   java.lang.Comparable

  Enclosing class:
  :   ConstrainedKMeans

  ---

    

  ```
  public static class ConstrainedKMeans.bucketInstance
  extends java.lang.Object
  implements java.lang.Comparable
  ```

  Class for representing an instance inside a bucket

- - ### Constructor Summary

    Constructors

    Constructor and Description

    `ConstrainedKMeans.bucketInstance()`
  - ### Method Summary

    Methods

    | Modifier and Type | Method and Description |
    | --- | --- |
    | `int` | `compareTo(java.lang.Object ci)` |
    | `double` | `getDistance()` |
    | `double[]` | `getDistances()` |
    | `void` | `setDistance(double x)` |
    | `void` | `setDistances(double[] x)` Sets the distances to other instances |

    - ### Methods inherited from class java.lang.Object

      `equals, getClass, hashCode, notify, notifyAll, toString, wait, wait, wait`

- - ### Constructor Detail


    - #### ConstrainedKMeans.bucketInstance

      ```
      public ConstrainedKMeans.bucketInstance()
      ```
  - ### Method Detail


    - #### setDistances

      ```
      public void setDistances(double[] x)
      ```

      Sets the distances to other instances

      Parameters:
      :   `x` - distances


    - #### setDistance

      ```
      public void setDistance(double x)
      ```

      Parameters:
      :   `x` -


    - #### getDistances

      ```
      public double[] getDistances()
      ```

      Returns:


    - #### getDistance

      ```
      public double getDistance()
      ```

      Returns:


    - #### compareTo

      ```
      public int compareTo(java.lang.Object ci)
      ```

      **Specified by:**
      :   `compareTo` in interface `java.lang.Comparable`


- Overview
- Package
- Class
- Use
- Tree
- Deprecated
- Index
- Help

- Prev Class
- Next Class

- Frames
- No Frames

- All Classes

- Summary:
- Nested |
- Field |
- Constr |
- Method

- Detail:
- Field |
- Constr |
- Method
